# Quality Aspects of Insects as Food—Nutritional, Sensory, and Related Concepts

**DOI:** 10.3390/foods8030095

**Published:** 2019-03-12

**Authors:** Mohammed Elhassan, Karin Wendin, Viktoria Olsson, Maud Langton

**Affiliations:** 1 Department of Molecular Sciences, Swedish University of Agricultural Sciences, SE-750 07 Uppsala, Sweden; maud.langton@slu.se; 2 Department of Food and Meal Science, Kristianstad University, SE-291 88 Kristianstad, Sweden; karin.wendin@hkr.se (K.W.); viktoria.olsson@hkr.se (V.O.); 3 Department of Food Science, University of Copenhagen, Bülowsvej 17, DK-1870 Frederiksberg C, Denmark

**Keywords:** cricket, grasshopper, neophobia, protein, nutrition

## Abstract

In the search for another appealing source of future food to cover the increasing need for nutrients of a growing global population, this study reviewed the potential of insects as human food. Most previous reviews have dealt with insects as a group, making it difficult to evaluate each individual insect species as food because of the generalized data. This study assessed some common edible insects, but concentrated on mealworms. Insects, especially mealworms, have a similar or higher nutritional value than many conventional food sources. For example, the protein content of mealworm larvae is reported to be almost 50% of dry weight, while the fat content is about 30% of larval dry weight. Mealworms can be cooked by different methods, such as hot air drying, oven broiling, roasting, pan frying, deep frying, boiling, steaming, and microwaving. Oven broiling in particular gives a desirable aroma of steamed corn for consumers. Changes in the flavor, taste, and texture of mealworm products during storage have not been studied, but must be determined before mealworms can be used as a commercial food source. Factors controlling the shelf-life of mealworms, such as their packaging and storage, should be identified and considered with respect to the feasibility of using mealworms on a commercial scale.

## 1. Introduction

**Figure 1:**
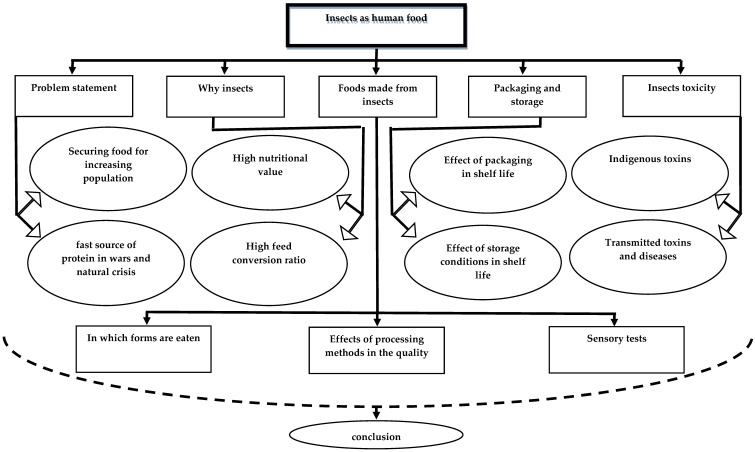
Flowchart of insects as human food literature review.

The global population is steadily increasing [1,2], making food security a crucial issue world-wide. Both the quantity and quality of food supply are of interest in the context of food security. All major nutrients are important, but protein is one of the main nutrients required by humans, and protein deficiency leads to severe illness. Protein from animal sources may be especially valuable, as it contains essential amino acids that are not found in many plant proteins. However, protein from animal sources is not always affordable for a large share of the global population [3]. Moreover, devastating wars are still occurring in various parts of the world, resulting in millions of people, especially innocent civilians, being displaced and often suffering from malnutrition due to a lack of food. During or immediately after wars, people need a fast source of protein, but conventional sources of protein (animals and plants) take a long time to become available. This means there is a need for fast protein sources that are nutritionally similar to protein from conventional sources. During the First World War, Germany used single-cell protein as the source of protein in animal feed, in order to meet human protein requirements [4]. However, direct consumption and excess consumption of single-cell protein by humans have negative effects on health, since it contains nucleic acids which cause the precipitation of uric acid in the human body, leading to gout and the formation of kidney stones. Another issue with the production of protein from animal sources is that it requires advanced resources, in many cases making it unsustainable for the environment [5]. Moreover, animal rearing for food is controlled by strict ethical rules and regulations, which can complicate the process. For the above reasons, an alternative source of protein is needed to assist in assuring food security for the increasing global population. One such source could be insects. The aim of this literature review was to examine the use of insects as a sensory appealing food and to identify knowledge gaps in this area (Figure 1). Although it is commonly claimed that insects are a non-traditional food, more than two billion people in 113 countries generally eat insects directly or use them as food ingredients [6,7,8]. It is estimated that there are up to 1900–2000 species of edible insect [6,9]. The tradition of eating insects is very old, e.g., there is evidence that insects were eaten during pre-historic times, forming an important source of protein [10,11].

## 2. Nutritional Value of Insects

Insects have been proven to have high nutritional value, especially the protein fraction in terms of adequate amino acid composition [12]. The amino acid composition in the larvae of yellow mealworm (*Tenebrio molitor*) is particularly favorable [13]. A study investigating four insects commonly eaten in Nigeria (African palm weevil, coconut palm rhinoceros beetle, caterpillar, and termite) found that the essential amino acids lacking in cereal protein, i.e., lysine and methionine, were present in relatively high amounts in these insects [14]. The overall protein, fat, and mineral content in insects has been investigated in many studies [15,16,17,18]. It has been found that the protein content ranges from 40 g/100 g to 75 g/100 g, the fat content from 7 g/100 g to 77 g/100 g, and the mineral content from 3 g/100 g to 8 g/100 g on a dry weight (DW) basis [19]. A study by Zielińska and co-authors found that the protein, fat, and mineral content of mealworm larvae was around 52 g, 24 g, and 1 g per 100 g dry sample, respectively [20]. A more recent study by Zhao and co-authors found that mealworm larvae contained about 51% protein, 32% fat, and 5% ash on a DW basis [13]. 

Furthermore, studies have shown that some insects have high concentrations of lipids. For example, the lipid percentage of yellow mealworm larvae was 27.4% [21]. This is why insects may be used to enrich food with lipids. When 20% grinded yellow mealworm larvae was mixed with wheat flour to make extruded cereals, the final product improved in terms of lipid content from 0.9% to 5.4% [21]. Insects can have high levels of unsaturated fatty acids, which needs to be considered during their processing and storage [13]. With regard to vitamins, insects in general have been found to have low percentages of retinol but to be rich in others such as riboflavin, pantothenic acid and biotin [18]. Folic acid, in some cases, has also been found in high amounts [18].

**Figure 2:**
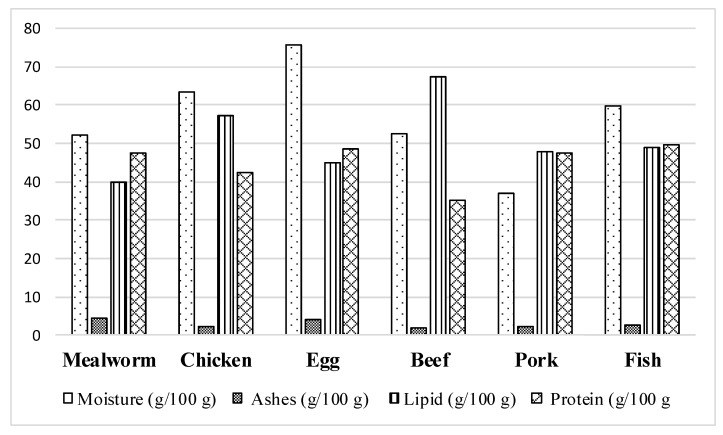
Comparison of the nutritional content of yellow mealworm larvae and conventional foods. Ashes, lipids and protein values based on dry weight. Source: Based on data from Alves et al. (2016).

Regarding the nutrient content, insects such as mealworms, but also other species, have the potential to complement and/or replace conventional foods, as they exhibit favorable nutrient profiles [20]. For example, the protein content of the grasshopper (*Schistocercagregaria*) is reported to comprise 76% of DW and that of tropical house crickets (*Gryllodessigillatus*) around 70% of DW, which is similar to the protein content of whey (~87% of DW) and chicken eggs (~82.1% of DW) [22]. In terms of the essential amino acids methionine and cysteine, house crickets contain the daily intake recommended by World Health Organization (WHO) [20]. Iron and zinc are very important micronutrients for human nutrition and insects are rich in minerals. For example, grasshoppers contain about 8 mg/100 g iron, which is higher than the iron content in beef (~6 mg/100 g). Fat content has a direct impact on the total energy of food. Whole milk powder contains about 26% fat [23]. Mealworms, which contain around 25% fat [24], can thus be considered a rich source of fat, and therefore of energy (Figure 2). 

## 3. Sensory Aspects

Nutritional values are of high importance, but for steady intake a food has to be acceptable from a sensory perspective. Use of insects as food is not common in the Western world, so consumers need to be convinced not only by their nutritional benefits, but also by their tastiness and general sensory appeal [25,26]. In cultures where they are eaten, most food insects are considered a delicacy, but they do not all taste the same and different species have the potential to serve different gastronomic functions [27]. In their book ‘On Eating Insects’, Evans et al. [27] compiled information on some edible insects and descriptions of their taste/flavor (Table 1). Books containing recipes for cooking insects have been published (e.g., Van Huis et al. [28] and Evans et al. [27]), but there is still a lack of information on how different insects react as ingredients and how a dish is tasting.

One factor to be taken into consideration is that most insects contain unsaturated fat. It is well known that unsaturated fatty acids can easily become rancid and that anti-oxidative processes and ingredients can delay rancidity development [30]. A recent study showed that rancidity had a negative impact on eating quality [31].

## 4. Insect Foods and Its Acceptance

Insects are prepared and eaten in different forms (Table 2) such as

*a. Whole insect*

Some insects, such as crickets, can be eaten whole after cooking [32]. Mealworm larvae may also be eaten whole after boiling [33].

*b. Dried and milled*

**Figure 3:**
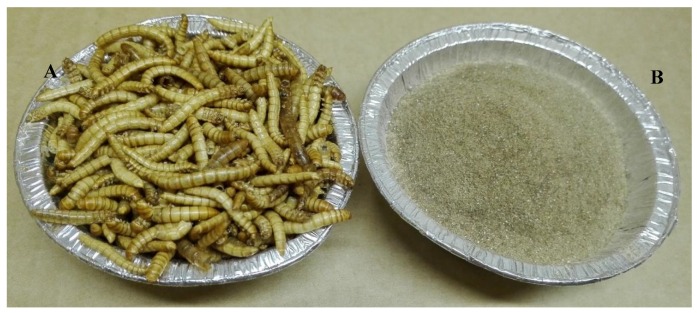
Yellow mealworm larvae (**A**) freeze-dried whole and (**B**) milled and freeze-dried. Photo by Xue Zhao, SLU, Sweden.

Insects can be also be dried and milled (see Figure 3), and then added to different types of food products, e.g., frankfurters, to enrich them in protein or other nutrients [34]. 

*c. Added fresh to food products after killing, without drying*

Processed foods such as burgers can be prepared from fresh mealworms by adding them whole to beef [33].

*d. As extracted nutrients*

The nutrients in insects can be extracted and added to foods. For example, Zhao et al. [13] extracted and characterized mealworm protein for the purpose of adding it to food products in order to enrich them with protein.

**Table 2:** Summary of some insect species that are eaten as food.

Insect	Commonly Considered as	Where EATEN Locally—Examples	Method of Preparation	Part of Insect Common LY Eaten
Mealworm	Cereal grain pest [35]	Africa, Asia, the Americas, Australia [36]	Stir-frying, grilling [37]	Whole larvae [38]
Cricket	Disgusting [39]	Asia (Thailand), Africa (Zimbabwe) [40]	Deep-frying [40]	Whole insects [40]
Grasshopper	Agricultural pest [41]	Africa (Angola), Asia (Japan), Latin America (Mexico) [40]	Frying [42]	Whole adult insect after removing wings [43] (wings and legs are removed)
Termite	Wood environment pest [44]	Sub-Saharan Africa (Uganda) [38]	Can be eaten raw [38]	Whole insect [45]

A study analyzing the nutritional value of yellow mealworm larvae found that it had good protein quality in terms of amino acids [46]. However, the acceptance of eating insects can be a challenge among consumers, especially in Western countries where insects are considered disgusting [8], and commonly associated with diseases and treated as pests [47]. This may be attributable to neophobia, a fear of eating novel foods. In the Western world, the aversion to eating insects is often culturally conditioned and based on neophobia and/or the perception that insects are disgusting, rather than on actual sensory experiences [48,49]. This means that finding enough people to participate in sensory tests of food made from insects can be a challenge in itself. Thus sensory tests may need to be performed using subjects whose culture involves eating the insect species to be tested, because they will not have a phobia about eating the insects that could affect the results of the sensory test. Some studies have conducted sensory evaluations on foods made from yellow mealworms to test the acceptability [33,34,46], but these studies are limited. An early study showed that the texture and flavor of a tortilla made from maize supplemented with yellow mealworm larvae powder was acceptable to consumers, so the inclusion of yellow mealworms in other types of food may be a promising option [46].

Food is sometimes enriched by adding only the extracted protein from mealworms [13]. However, in the study by Aguilar-Miranda et al. [46] cited above, whole mealworm larvae powder was added. A study in Belgium evaluated the taste, appearance, flavor, and overall liking of burgers made from beef, mealworm/beef, lentil, or mealworm/lentil and found that the burger made from mealworm/beef was acceptable in terms of the taste, being second to beef in terms of acceptability [33]. In a study in Korea, the flavor and taste of muffins enriched with up to 8% mealworm powder were found to be acceptable to tasters [34].

Other studies have added some flavor, such as chocolate, to mealworms and have found that this improves the appeal of mealworm-based products [50]. A study in the Netherlands found a correlation between acceptability in terms of texture and flavor, and willingness to buy foods made partly from mealworms [51]. That study also found that the willingness to buy meatballs made from mealworm decreased after tasting. This may indicate that theoretical acceptability in terms of a willingness to buy insect-based foods, without actually tasting the insect product, may not always reflect the real outcome. This means in turn that sensory tests of food made from mealworms are required to reveal the true acceptability. The type of food matrix and the percentage of mealworm added are important factors controlling consumer acceptability. For example, in a study in which the pork in Frankfurters was replaced with mealworm, replacing up to 10% of pork was acceptable, but a higher percentage replacement rate resulted in increased off-flavor and reduced juiciness of the product, which was deemed undesirable [34]. Thus, the addition of mealworms does not always increase the desired characteristics of food products.

### Neophobia of Eating Insects as Foods

In a study involving an internet-based questionnaire, Wendin et al. [26] sought to determine consumer acceptance and neophobia of eating insects as food. The study showed that the acceptance of eating food enriched by insect protein powder was significantly higher than the acceptance of eating food with added whole insects. Similarly, Zhang [52] found that the acceptance of eating insects is higher among Swedish consumers if the insects are not visible. Furthermore, the acceptance of eating insects has been shown to be higher among people with previous experience of eating insects and among those with low neophobia [53,54]. This may be because the more familiar a food is, the more it is liked and accepted [55]. Wendin et al. [26] found that interest in buying insect protein powder was significantly higher than in buying whole insects. Both food texture and the extent to which foods are similar to living animals are important for the perceived “disgustingness” of various foods [56]. It was found that 16% of the people involved in the survey by Wendin et al. [26] were neophobic. The very important implication from that study is that culinary approaches and how the food is served to consumers will play a very important role in the future use of insects as human food. 

Providing information about the benefits of eating insects can increase long-term intention by consumers to eat insects and this intention can carry over to behavior [57]. 

## 5. Instrumental Sensory Evaluation

Some sensory evaluations can be performed using instruments. For example, Azzollini et al. [21] measured the texture of extruded snack foods made of wheat flour enriched with mealworm larvae powder using a texture analyzer. They found that addition of mealworm larvae increased the maximum force, which indicated an increase in the desirable crunchy texture. Crunchiness is a preferred characteristic in extruded food. Few other studies have investigated the texture, taste, and flavor of mealworms as food. 

## 6. Effect of Food Processing Methods on Products Made from Insects

Although not all types of possible food products have been investigated, there is evidence that food processing method affects the quality of products made from yellow mealworms. For example, in a study in which barrel temperature was increased during the processing of extruded insect-enriched snacks, it was found that this affected the texture of the snacks [21]. Texture was measured as the maximum compression force (Fmax) (N) and it was found that when maximum barrel temperature was 120 °C at screw speed 400 rpm, Fmax was 281 N, but when the temperature increased to 160 °C, Fmax decreased to 171 N. This means that there is an inverse relationship between barrel temperature and the crunchy desirable texture of extruded mealworm-enriched snacks. Mealworm larvae are officially recognized as food ingredients by government authorities in some countries, such as Korea [58]. Cooking conditions for mealworm larvae affect their quality as food in terms of physical properties and sensory characteristics. The microwaving of yellow mealworm larvae leads to the highest values of some physical parameters, such as texture, hardness, and fracturability, while the highest values of adhesiveness, springiness, and chewiness have been found to be associated with boiling the larvae [58]. The L*a*b value of a food is measure of the color, where L* indicates lightness, a* is the red/green coordinate, and b* is the yellow/blue coordinate. Baek et al. [58] recorded the L*a*b value for boiled and steamed mealworm larvae and found that oven broiling led to the highest redness and yellowness of the mealworm larvae, measured as a and b values, respectively. 

In terms of the effect of cooking methods on sensory properties, it has been found that boiling and steaming mealworms keep the larvae similar in appearance and shape to the fresh larvae [58]. Boiling and steaming also maintain the size of mealworm larvae better than other methods of cooking. Larvae cooked by boiling and steaming are characterized by the flavor of steamed corn, canned pupa, and boiled mushroom (see Table 1). However, oven-broiling is reported to be the best cooking method, as it results in a desired aroma of mealworm oil, plus an aroma of seafood, sweetness, and roasted sesame [58]. Sensory evaluations of mealworm larvae cooked by hot air drying and oven broiling give higher scores in terms of hardness and crispiness as texture parameters compared with other methods, while larvae cooked by steaming and boiling are rated higher in juiciness [58]. Thus, the oven broiling method can be recommended for mealworm larvae, due to its production of the desired aroma and flavor for consumers.

## 7. Farm Rearing of Edible Insects

**Figure 4:**
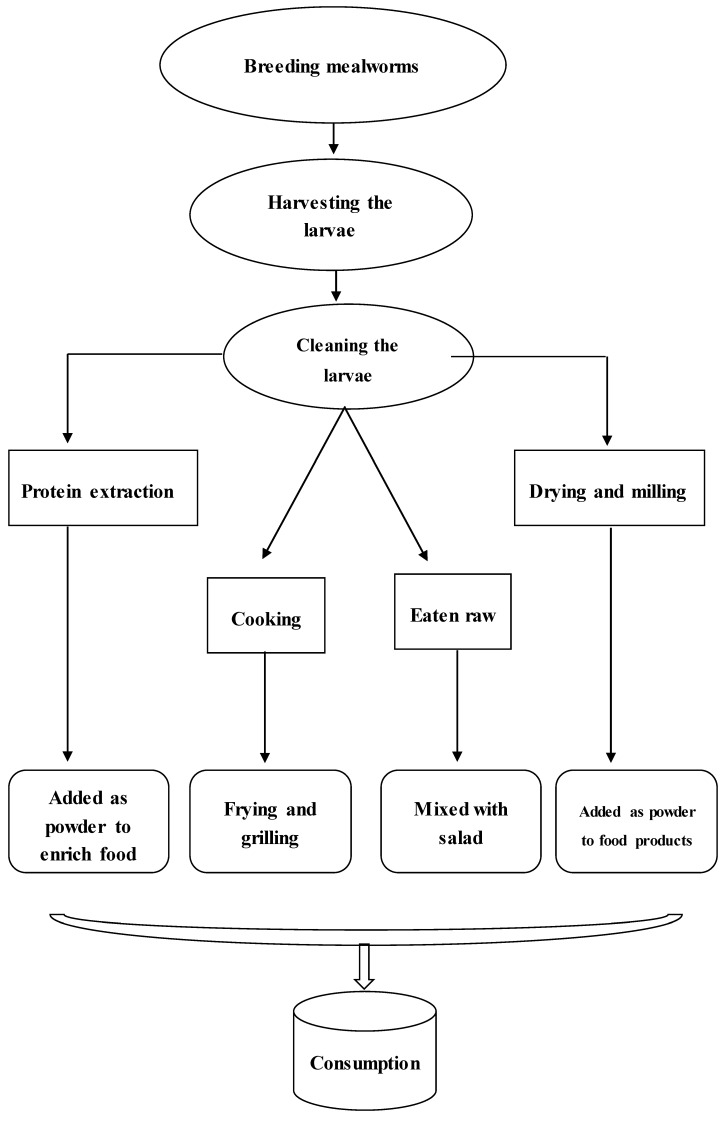
Main steps from farm to consumer in the use of mealworms as human food.

Since one of the reasons for using insects as food is their high feed conversion ratio and fast production of protein mass compared with conventional protein sources (animals and plants) [59], the rearing of insects is a critical step (Figure 4). The economic viability can determine production of farmed insects, so finding cheap methods to feed and rear edible insects is an important issue in the literature. Varelas and Langton reviewed the use of forest biomass waste as feed for edible insects [60]. However, there are other possibilities and approaches for rearing edible insects. Based on the part of the insect that is eaten, the methods used for rearing may differ. In the past and in some cases even now, people were dependent on wild insects for some type of food, e.g., most humans are familiar with reared insects such as honeybees, where the process of rearing is called apiculture. In this case, rearing does not target the size of the bees, but rather the quantity and the quality of the honey [61]. In the case of crickets, as they are usually consumed as whole insects they must be large in size [62]. Megido et al. [33] tested different types of rearing in terms of feed components and compared the quality of the walker cricket (*Teleogryllustestaceus*) as food. In particular, they tried to use local sources of cricket feed. They found that the highest biomass was obtained using broiled cricket feed, but that biomass also high with feeds containing cassava leaves and brown rice. In terms of the cost, the feed that contained cassava leaves were the cheapest [33]. However, the influence of the types of feed on an insect’s nutritional quality was unclear [63].

The economic and environmental aspects are important factors in using insects as food. The high feed conversion ratio of insects [59] should be considered an advantage in providing food from insects, with low feed consumption compared to the conventional meat protein production from animal origin, and therefore low costs of production [8]. Beef animals need at least 20 kg of corn and soybean to produce 1 kg of meat [64], while insects such as crickets require only about 2 kg feed to give 1 kg of body mass [8]. In terms of water consumption during rearing, insects consume less water than animals. Less consumption of water is positive for the environment. It has been estimated that 8% of global water resources consumed for livestock production [65]. Compared to animal production, the production of feed for insects competes less with human food production. The feed of animals can be supplemented by fish meal, bone meal, blood and even plant protein from sources such as sunflower, soybean and cotton seedcake [66,67]. There are studies explaining that insects are considered highly environmentally friendly, which is attributed to the lower levels of greenhouse gases such as methane, nitrous oxide and carbon dioxide produced by insects compared to cattle [68]. However, according to Berggren at al. (2019) there is more to be taken into account than a comparison of feed-conversion in order to develop the rearing of insects into a sustainable business [69].

## 8. Packaging and Storage

Packaging is a critical factor during the storage of insects. The effect of temperature on cricket powder flavor and acidity during storage has been investigated [70]. The results revealed that cricket powder stored at 25 °C and 35 °C did not exhibit an increase in off-flavor after six months. However, when the cricket powder was stored at 40 °C, it showed a significant increase in off-flavor compared with the initial value. The acidity value of cricket powder, monitored during storage, showed a negative relationship with storage temperature [70]. This decrease in the acidity of cricket powder due to increased temperature can be attributed to the oxidation of fat. Thus, the sensory attributes of cricket powder seem to be stable at 25 °C and 35 °C, while inferior sensory evaluation can occur at 40 °C [70]. Regarding microbial growth during the storage of cricket powder, aerobic and coliform bacterial growth was not detected during that six-month study. For a type of grasshopper (*Ruspolianitidula*), it has been found that vacuum packaging can maintain sensory acceptability, and thus improve/enhance storage stability at ambient temperature, and that the quality can be further improved by low-temperature storage [66].

Oonincx and De Boer [71] concluded that, at the time of their study, there was insufficient evidence available for the standardization of storage conditions for mealworms. The studies performed since 2012 have focused solely on microbial growth during the storage of mealworms. For example, Stoops et al. [72] examined the effect of different storage conditions on microbial growth in mealworms and concluded that replacing air in the storage environment with carbon dioxide and nitrogen reduced microbial growth during storage. Musundire et al. [73] found aflatoxin in stinkbug packed in recycled grain containers, which are traditionally used to package these insects, and attributed this to cross-contamination from the packaging. However, there is still a lack of published data on the effect of packaging type on the shelf-life of edible insects prepared as human food.

## 9. Toxicity and Allergy to Insects

Insects have long been eaten in many parts of the world by local people in different areas, but cases of poisoning and allergic symptoms have been recorded. In south-west Nigeria, cases of seasonal ataxic syndrome after consumption of silkworms (*Anaphevenata*) have been reported [74]. Ataxia is a neurological defect resulting in loss of full control of bodily movements and one of the causes of ataxia is thiamine deficiency. The larvae of *Anaphevenata* contain the relatively heat-resistant enzyme thiaminase and, on consumption of silkworms, the thiaminase can break down the thiamine in the human body, resulting in acute ataxia [75].

Microbial growth in insects can result in the transmission of toxins by insect foods. The initial total viable count of microbes in fresh mealworms after rearing is reported to be 7–8 log cfu/g [76]. This high microbial level in mealworms should first be reduced and then the further growth of microbes should be prevented, in order to avoid any possibility of food poisoning.

It has been reported that people who suffer from some type of food allergy, for example to shellfish, may be susceptible to health risks when eating insects [77]. This means that foods containing insects must be labelled in order to protect those suffering from food allergies. 

## 10. Conclusions

There have been relatively few studies on insects as human food in general and the evaluations and comparison of edible insects as food reported to date do not give conclusive results. For example, there may be variations in the sensory evaluations of edible insects due to differences in how the studies are conducted. Therefore, detailed and consistent studies targeting certain individual insects, preparation, storage and other aspects are needed to give a clearer picture of the use of insects as human food. There are currently no published studies dealing with factors influencing the oxidation of mealworm fat and protein during storage. The high nutritional value of insects can be considered the main factor justifying the use of insects as a human food. However, sensory appeal may be the key to insects being valued as a pleasurable component of a meal. Mealworms can be cooked in different ways and has the potential to be commercialized as a human food, but more studies are needed on related aspects such as production economics, sensory properties, optimum storage, and potential toxicity.

## Figures and Tables

**Table 1:** Some edible insects and descriptions of their sensory properties.

Insect (English)	Insect (Latin Name)	Sensory Description
**Mealworm**	*Tenebrio molitor*	Nutty, umami. Intense aroma of cereal, nuttiness, and wood, a less pronounced aroma of broth. Intense flavor of nut, cereal, and umami and slightly less intense flavor of vegetables and Maillard reaction products. Brittle texture.
	*Alphitobiusdiaperinus*	Distinct aroma of cereal and nuttiness, notes of broth. Intense flavor of umami, nuttiness, and cereal. Less intense notes of vegetables and Maillard reaction products. Crumbly texture.
**Cricket**	Sub-order Ensifera *Achetadomesticus*	Umami, popcorn, chicken, mild, creamy. Pronounced aroma of broth, nuttiness, and cereal, with notes of wood. Intense flavor of umami and vegetables. Apparent note of nuttiness and, a bit less pronounced, cereal and Maillard reaction products. Crumbly texture.
**Grasshopper**	Sub-order Caelifera *Locustamigratoria*	Shrimp. An intense aroma of cereal, wood, and nuttiness. Notes of broth and fruit. The flavor has an intense umami and vegetable character, combined with nuttiness and cereal, and notes of Maillard reaction products and relatively low saltiness. Crusty, hard, and coarse texture.
**Red wood ant**	*Formica rufo*	Intense sourness, lemon
**Black ant**	*Polyrhachisvicina*	A distinct aroma of soy sauce and broth, emergent note of berries and less so fruit. Intense sour flavor with a pronounced Maillard reaction product character. Notes of umami, nuttiness, and bitterness. Crusty, coarse, grainy, and hard texture.
**Jet ant**	*Lasiusfuliginosus*	Mild acidity, kaffir lime
**Termite**	*MacrotermesbellicosusMacrotermessubhyalinusNasutitermestriodiae*	Crunchy, nutty, fatty, savory. A pronounced aroma of nuttiness and broth, with notes of cereal, wood, and soy sauce. Intense flavor of Maillard reaction products, salt, and nuttiness. Flavor notes of umami and cereal, and a relatively mild vegetable flavor. Texture characterized by coarse grain and brittleness.

Refs. [27,29].
